# The trochlear isometric point is different in patients with recurrent patellar instability compared to controls: a radiographical study

**DOI:** 10.1007/s00167-017-4740-2

**Published:** 2017-12-04

**Authors:** Tulio Campos, Akash Soogumbur, Iain R. McNamara, Simon T. Donell

**Affiliations:** 1 Department of Orthopaedics, Universidade Federal de Minas Gerais, Belo Horizonte, Brazil; 2 Department of Orthopaedics, Norfolk and Norwich University Hospital, Norwich, Norfolk, NR4 7UY UK; 3 Norwich Medical School, Faculty of Medicine and Health Sciences, University of East Anglia, Norwich, NR4 7TJ UK

**Keywords:** Medial patellofemoral ligament, Femoral tunnel, Isometry, Schöttle’s point, Trochlear dysplasia

## Abstract

**Purpose:**

The purpose of the study was to investigate the theoretical isometric point based of the curve of the femoral groove and relating it to the origin of the MPFL femoral tunnel on lateral radiograph by comparing a patellar instability cohort with a control cohort.

**Methods:**

From a Patellar Instability database the radiographs of 40 consecutive patients were analysed to define Schöttle’s point, and the arc of the circle of the trochlear groove. A comparison population of 20 radiographs from comparable patients with tibiofemoral joint disorders was used as a control. The distance from Schöttle’s point to the most anterior part of the groove (extension) was also compared to the distance to the distal end of the roof of the notch (flexion).

**Results:**

The trochlea was circular in the controls but not the Patellofemoral Instability cohort where trochlear dysplasia is usually present. The difference between the extension and flexion length was a mean of − 2.0 ± 0.5 mm in the controls and + 6.0 ± 0.5 mm in the patellofemoral cohort. In neither cohort did the centre of the circle correspond to Schöttle’s point. The extension distance correlated with the boss height.

**Conclusions:**

The dysplastic trochlea is not circular and the centre of the best matched circle was different to the control trochleae which were circular. The circle centres did not correlate with Schöttle’s point for either cohort, and was more proximal in the Patellofemoral Instability cohort.

**Clinical relevance:**

For the MPFL to have equal tension throughout flexion within the groove, the length should not change. In normal knees the MPFL does not behave isometrically. The change in length, as measured from Schöttle’s point to the trochlea, was greater for patellofemoral instability patients explaining why an isolated MPFL reconstruction in the presence of severe trochlear dysplasia risks poor outcomes.

*Level of evidence* III.

## Introduction

Reconstruction of the medial patellofemoral ligament (MPFL) has become a popular operative strategy for managing recurrent patellar dislocation, for which a number of techniques have been described [13]. The importance of the MPFL in providing stability to the patella and restraining lateral displacement of the patella from the femoral sulcus is well recognised [1]. The origin and insertion of the medial patellofemoral ligament are well described, and the concept of isometricity for achieving proper clinical function has also been proposed [19]. In fact current dogma states that unless the femoral tunnel is in the correct position in a medial patellofemoral ligament (MPFL) reconstruction then a poor result is more likely [2, 20]. Up to 15% of MPFL revisions have been reported as due to poor femoral tunnel position, and 21% due to excessive graft tensioning [12]. The precise point for the origin of the femoral tunnel is debated but tends to correlate with the origin of the normal MPFL [2, 5, 10, 18, 25] with the consensus being that this is 10 mm distal to the adductor tubercle. Since, in the sagittal plane, the normal trochlea is an arc of a circle [9], the centre of the circle identifies an isometric point where the length remains constant through the first 90° of knee flexion. If this point was used for the femoral tunnel position of an MPFL reconstruction then the ligament would behave isometrically. Even so, the conventional radiographic points used to define the femoral anatomical placement only approximately correspond to the anatomical position and should not be used as the sole basis for the femoral attachment location, although this work was done on normal cadaveric knees [15].

The problem with relying on anatomical landmarks for the origin of the MPFL is that all the work has, as stated above, been based on normal knees. However, the path of the patella during knee flexion changes completely in the presence of trochlear dysplasia (TD) as the proximal part of the groove is anterior to the path of the normal groove. It is logical to expect that the more severe the dysplasia the further from the norm the patellar movement will be and the more anisometric an MPFL reconstruction will be if the standard femoral tunnel position is chosen. This has been confirmed clinically in that MPFL reconstruction alone does not work well in patients with significant TD [6, 8, 11, 12].

The purpose of this study was to identify the radiographical “isometric point” of the trochlea from a cohort of patients presenting with patellar instability and compare this in a control population with tibiofemoral disorders and also with a recognised femoral tunnel position, the Schöttle’s point [18]. The hypothesis was that the radiographical “isometric point” in patients presenting with patellar instability is different to that of a control population and that this would be anterior and proportional to the degree of trochlear dysplasia as measured by the boss height [14].

## Materials and methods

Patients with recurrent patellar dislocation were identified from the institution’s Bluespier Data Management software (Bluespier International); within it is a dedicated Patellofemoral Database. From this, the first 40 patients with perfect pre-operative lateral plain radiographs (where the posterior femoral condyles overlap precisely) were selected. The plain radiographs were stored on the hospital Picture Archiving Computer System (PACS) using the standard measurement tools available on the PACS software (Fuji Synapse). This cohort was called the PFI (patellofemoral instability) cohort. The patients’ basic demographics were; mean age was 26-years-old (range 17–61-years-old), gender 19 male and 21 female, side 21 right and 19 left.

A comparison Control cohort was identified from patients presenting to a Knee Clinic and selected for arthroscopic surgery for tibiofemoral disorders; ACL reconstruction or meniscal repair. Twenty consecutive patients were selected and images retrieved as above. The mean age was 26-years-old (range 15–42), 11 were male and 9 female, with 12 right knees and 8 left.

### Radiographical analysis

**Fig. 1:**
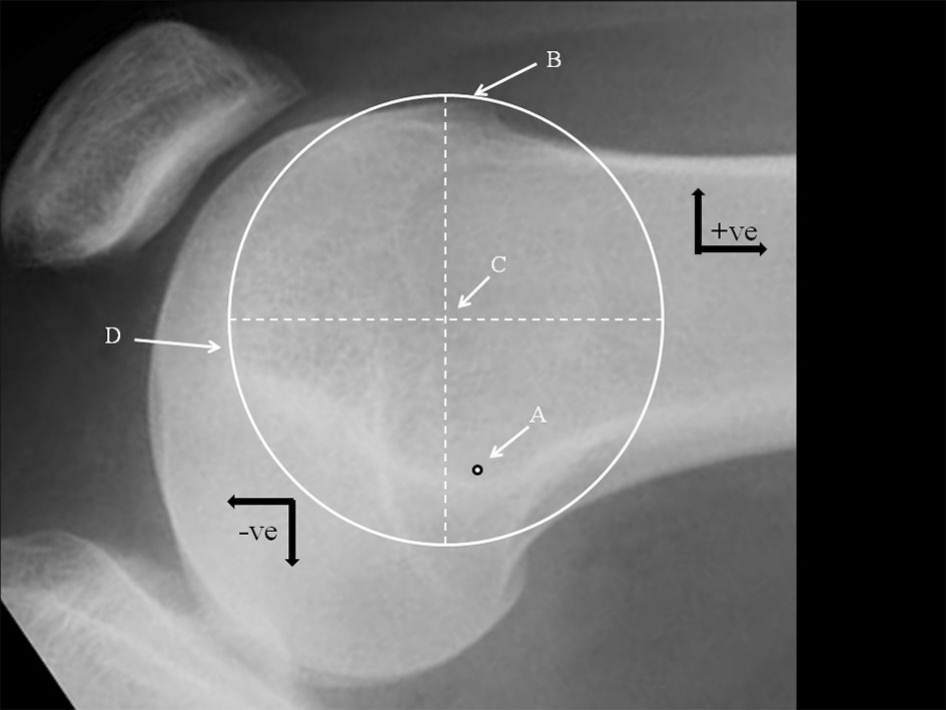
Lateral radiograph of the knee showing: *A* Schöttle’s point. *B* Most anterior point of the groove (also the point where the boss height is measured). *C* Isometric point (centre of the *circle* that includes the groove). *D* Most distal point of the groove. Black arrows indicate convention chosen for defining relative position of a point

The radiographical analysis began by identifying the femoral tunnel position as defined by Schöttle [17] and marked ‘A’ (Fig. 1). Schöttle’s point lies just distal and anterior to a line drawn at right angles to an extension of the posterior femoral cortical line. The boss height was then measured [14], which is the distance between the extension of the anterior femoral cortical line and the right angle to the most anterior point of the groove. Using the ellipse tool available on the PACS program, and on the assumption that a dysplastic groove is an arc of a circle, a circle was created to match the groove. Circularity was confirmed by measuring and equalising the diameters at right angles. The centre of this circle was defined as the “isometric point” and marked ‘C’. The distance between A and C was measured and recorded. Using the anatomical axis (horizontal offset), the distance proximal (negative) and distal (positive) between A and C, and the right angle to this (vertical offset) were measured. The vertical offset was defined as positive if anterior and negative if posterior to the anatomical axis. No special scaling program was used to correct for any magnification errors and the results were measured in millimetres rounded to the nearest 0.5 mm. Two further measurements were taken, from point A to the most anterior point of the boss (B), and also from point A to the most distal point of the trochlea (D). The PFI cohort was further subdivided into those with a boss height < 5 mm and those whose boss height was ≥ 5 mm. A boss height of ≥ 5 mm has been defined as significant TD [6].

One author (Observer 1) undertook the measurements. Twenty patients were then randomly selected and the same author repeated the measurements 1 week later. These 20 were also analysed by a second author (Observer 2). The comparison cohort observations were undertaken by Observer 2.

Radiographic review does not require ethical approval in the UK. The research database has approval for use for research purposes and report writing by the Research and Development Department reference number 2011ORTH09L (142-10-11).

### Statistical analysis

The data were collected and stored on an Excel spreadsheet. Inter- and intra-observer differences were analysed as means and standard deviations. These data were compared by *t* test using Wizard for Mac version 1.8.16 since the data were continuous and normally distributed (confirmed by Shapiro–Wilk test) Comparisons were analysed between control and study cohorts. Simple regression analysis was undertaken for correlations between boss height and vertical or horizontal offset for both cohorts combined. Statistical significance was set at *p* = 0.05.

## Results

In all the parameters there were no statistically significant differences between the observers or within an observer (see Tables 1, 2). The control group was found to have grooves that matched the arc of a circle, but not in the PFI group where the dysplasia was significant.

**Table 1:** Intra-observer variability of the measures

	Observer 1 first (*n* = 20)	Observer 1 second (*n* = 20)	*t* test (*p* value)
AC distance (mm)	8.5 ± 4.0	9.5 ± 3.5	ns
Vertical offset (mm)	8.0 ± 3.0	9.0 ± 3.5	ns
Horizontal offset (mm)	0.5 ± 1.0	0.0 ± 3.0	ns
Boss height (mm)	5.6 ± 2.5	5.5 ± 2.5	ns
AD distance	30.5 ± 3.5	31.0 ± 4.0	ns
AB distance	37.5 ± 4.0	38.0 ± 4.0	ns
Circle radius	29.0 ± 4.5	29.0 ± 5.0	ns

**Table 2:** Inter-observer variability of the measures

	Observer 1 (*n* = 20)	Observer B (*n* = 20)	*t* test (*p* value)
AC distance (mm)	9.5 ± 3.5	8.0 ± 3.5	ns
Vertical offset (mm)	9.0 ± 3.5	7.0 ± 4.0	ns
Horizontal offset (mm)	0.5 ± 3.0	2.0 ± 2.5	ns
Boss height (mm)	5.5 ± 2.5	5.0 ± 3.5	ns
AD distance	31.0 ± 4.0	31.0 ± 3.5	ns
AB distance	38.0 ± 4.0	36.0 ± 5.0	ns
Circle radius	29.0 ± 5.0	29.0 ± 4.0	ns

The measurements and analyses for the whole cohort are shown in Table 3. It can be seen that the circle radius was larger in the PFI cohort reflecting the TD. The change in length between extension and flexion (AB minus AD distance) (Table 3) was − 2 mm in the controls, to + 5.5 mm when the boss height was < 5 mm, and + 7.0 mm when the boss height was ≥ 5 mm.

**Table 3:** Measurements and analyses of the relationship between the cohorts

Cohort	Control (*n* = 20)	PFI all (*n* = 40)	PFI boss height < 5 mm (*n* = 25)	PFI boss height ≥ 5 mm (*n* = 15)	Control with PFI *t* test (*p*)	Control with boss height < 5 mm *t* test (*p*)	Control with boss height ≥ 5 mm *t* test (*p*)	Boss height < 5 mm with boss height ≥ 5 mm *t* test (*p*)
Circle radius	25.5 ± 3.0	29.0 ± 4.5	28.5 ± 4.0	30.0 ± 3.5	< **0.01**	< **0.01**	< **0.001**	ns
AC distance (mm)	9.0 ± 3.0	9.0 ± 3.5	7.5 ± 3.5	10.0 ± 2.5	ns	ns	ns	**0.04**
Vertical offset (mm)	7.0 ± 3.5	8.5 ± 4.0	6.5 ± 4.5	9.5 ± 2.0	ns	ns	**0.03**	**0.02**
Horizontal offset (mm)	5.0 ± 2.5	1.0 ± 2.5	0.5 ± 2.5	0.5 ± 2.0	< **0.0001**	< **0.0001**	< **0.0001**	ns
Boss height (mm)	0.0 ± 2.0	5.0 ± 3.5	3.5 ± 1.5	7.5 ± 1.0	< **0.0001**	< **0.0001**	< **0.0001**	< **0.0001**
AD distance	34.0 ± 5.0	30.0 ± 4.5	29.5 ± 3.0	32.5 ± 3.0	< **0.01**	< **0.001**	ns	< **0.01**
AB distance	32.0 ± 4.0	37.0 ± 5.0	35.0 ± 4.0	39.0 ± 3.0	**0.0001**	**0.01**	< **0.001**	< **0.01**
AB distance minus AD distance	− 2.0 ± 0.5	+ 6.0 ± 0.5	+ 5.5 ± 0.5	+ 7.0 ± 0.5	< **0.0001**	< **0.0001**	< **0.0001**	< **0.0001**

Bold values indicate significant results

No correlation was found between boss height and the vertical offset (*R* ^2^ = 0.06, ns) but was for the horizontal offset (*R* ^2^ = 0.30, *p* < 0.0001) where the greater the boss height the more proximal the trochlear isometric point compared to controls. However the difference in length between AB and AD in the cohorts correlated to the boss height (*R* ^2^ –0.29, *p* < 0.3).

**Fig. 2:**
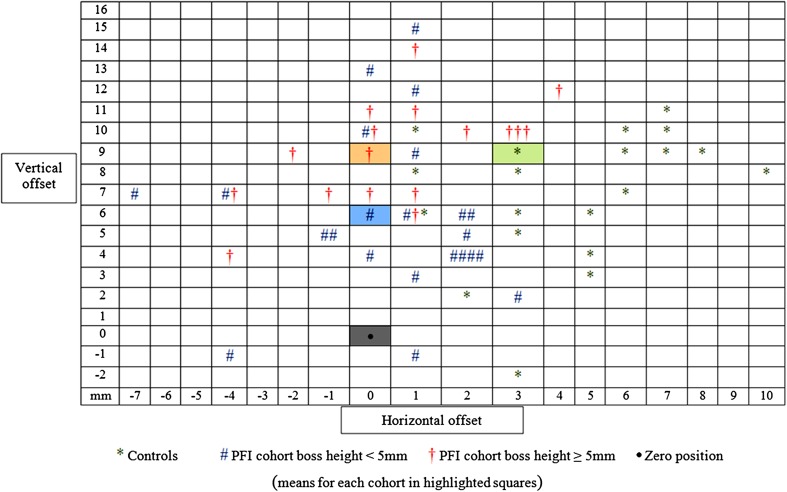
Scatter plot showing the position of the circle centres for each cohort relative to the MPFL origin

Figure 2 shows the scatter of the isometric points by cohort (controls, PFI with boss height < 5 mm, and PFI with boss height ≥ 5 mm).

## Discussion

The main findings of this study were that the dysplastic trochlea in PFI patients does not form a circle in the sagittal plane unlike normal controls. The centre of the best-fit circle (isometric point) does not correspond to the femoral origin of the MPFL with normal controls having the isometric point on average more distal, whereas in the dysplastic group the point tended to be proximal. The more severe the dysplasia, the more anterior the isometric point was relative to Schöttle’s point (Fig. 2). Furthermore, the variance in length when measured from Schöttle’s point was dependent on boss height with the change in length between extension and flexion of − 2 mm in the controls, to + 5.5 mm when the boss height was < 5 mm, and + 7.0 mm when it was ≥ 5 mm.

Since the origin of the MPFL does not match the isometric point in normal trochleae, then the MPFL cannot behave isometrically through the range of knee motion in the normal knee. This is well recognised; the ligament slackens in flexion where the patella is “captured” by the depth of the groove and the MPFL is not required to act as a check rein and stop lateral displacement [1]. The difference is greater in a PFI population and reflects the presence of TD. It should be emphasised that the isometric point of the trochlea is highly variable in any patient group.

**Fig. 3:**
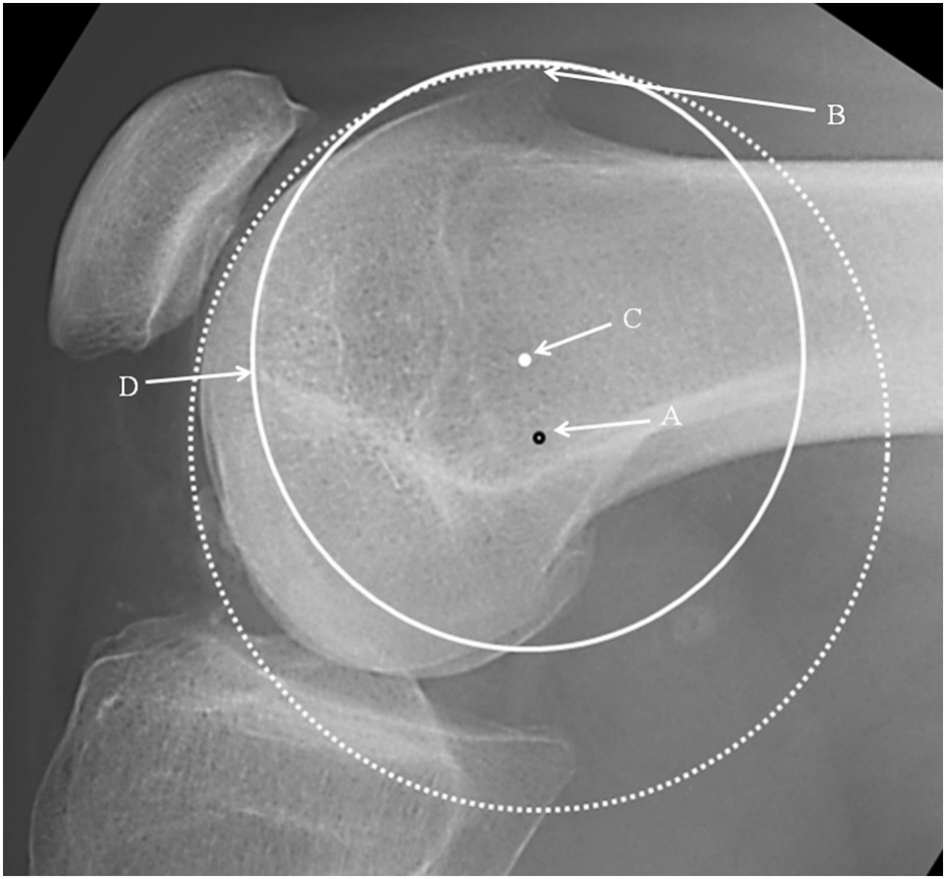
Lateral radiograph of the knee showing: Schöttle’s point: black rimmed spot (A). Isometric point: white spot (C). Dotted circle: centred on Schöttle’s point with the radius to the highest point of the boss (B). Complete circle: centred on the isometric point. Note that the dysplastic groove is not an arc of this circle. Point **D** is the distal end of the notch

**Fig. 4:**
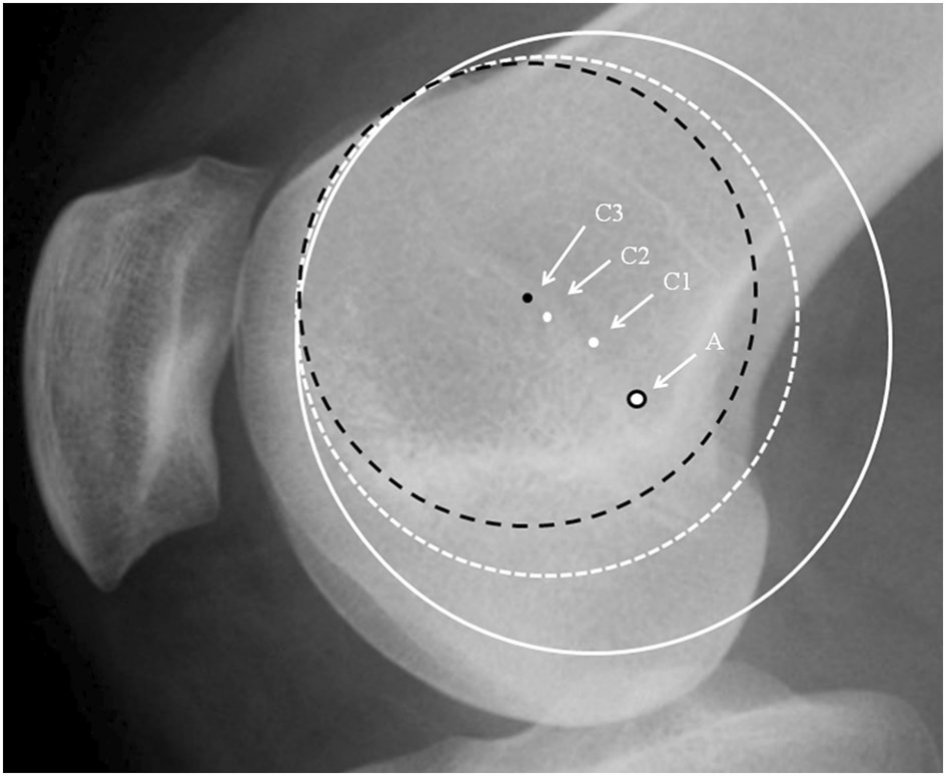
Lateral radiograph of the knee showing different circles depending on how the observer chooses the best circle-fit to the dysplastic trochlea curve: Schöttle’s point: black rimmed spot (A). Centres of white circles: white spot (C1) solid circle and (C2) dashed circle. Centre of black circle: black spot (C3)

It was noticeable that when applying a circle to the groove line in the PFI cohort, the circle could be placed to touch two points on the groove, e.g., B and D (Fig. 3) or the circle could match much of the groove (Fig. 4). In the control cohort the groove matched a circle. In Fig. 3, for a patient with TD, it can be seen that the isometric point is anterior to Schöttle’s point, and the radius of the circle from Schöttle’s point to the apex of the boss is longer than that for the best-fit circle. This would result in a significantly anisometric graft if the Schöttle’s point was used as the femoral tunnel position. In Fig. 4, in TD, isometric point moves anteriorly as the circle is made smaller. This implies that there is potential for significant error in the isometric point position in TD. A much more complex methodology is needed requiring 3D reconstruction and analysis of the tracking of the patella to define the femoral tunnel position in the presence of TD. This is not appropriate for the surgeon in a clinic. Interestingly, using 3D CT scans in different degrees of knee flexion, Blatter et al. [4] only found one isometric point in 10 normal knees when measuring the MPFL length with a maximal length difference during knee flexion of 10 mm. They also noted that for most, the optimal tunnel position was slightly anterior to Schöttle’s point. It would be interesting to know how this correlated with the lateral plain radiograph.

Ziegler et al. [26] emphasised the importance of a true lateral radiograph using normal cadaveric knees. The position of the MPFL tunnel was 4 mm away from the true MPFL origin as defined by dissection, worsened by even 2.5° rotation of the image. However, one needs to question the clinical relevance of a 4 mm mal-location if the tunnel is fixed with a 6 or 7 mm screw. In significantly dysplastic knees there is no certainty where the femoral origin of the MPFL is at operation, and malposition using fixed points on a radiograph is highly likely. A recent study by Alfonso-Sanchis et al. [16] looked at their clinical and radiological results of a cohort of patients who had undergone an MPFL reconstruction. Their operative technique is to place the femoral tunnel in a non-anatomical position. Of note this is anterior to Schöttle’s point, and is in keeping with the results of our study. We could have chosen any fixed point for the femoral tunnel apart from Schöttle’s point [25], since the message is to consider the relative length between that point and the most anterior point of the groove (for the length in extension), and to the distal end of the roof of the notch (for the length in flexion). Alfredo-Sanchis et al’s [16] showed the distance between the femoral origin and patellar insertion (which they termed “length”) was at a maximum at 0° flexion and measured a mean of 52 mm ± 5 mm, which corresponds to the maximal boss height. The distance between the actual femoral tunnel and the anatomical position was not reported, but the clinical graft distance was isometric between 0° and 30°, and the graft became lax after 60°. In our study the absolute numbers are likely to be inaccurate, but the surgeon can consider the relative lengths and where they might want the tunnel to go using the intra-operative bony landmarks. Ultimately the position is decided intra-operatively [16].

The assumption in this study is that, in the sagittal plane, the path of the patella is defined by the shape of the trochlea, and that the patella is at a normal height. Given that the trochlea is highly variable in patients with patellar dislocation since the majority have TD, then this explains why significant TD is a risk factor for failure of isolated MPFL reconstruction [8]. Having said that it also needs to be recognised that, clinically, if a graft is fixed with a 6 mm interference screw in a 6 mm tunnel, then the final graft position will be 3 mm away from the guidewire position, and could finally rest anywhere around the circle of the tunnel. Looking at Fig. 2 and considering a circle of radius 3 mm around the zero point, then most of the circle centres were outside. However, MPFL is a successful operation [18]. This implies that precise positioning (within 3 mm at least) is not the essential factor. A recent study by Hiemstra et al. [7] of a cohort of 155 patients who had undergone an MPFL reconstruction showed that the post-operative outcomes did not correlate with the femoral tunnel position with respect to Schöttle’s point. Of note, the femoral tunnel position was decided intra-operatively with a technique that aimed to have the graft tight in extension and lax in flexion. The distance between the femoral tunnel and Schöttle’s point had a mean of 6 mm ± 4 mm, although they did not report the direction of the difference. Logic dictates that the graft should be inserted and fixed without tension when the patella sits at the point of maximum distance from the femoral tunnel. As a result, if the Schöttle’s point is used and there is a TD with a boss height > 5 mm, then the graft should be fixed at around 20° flexion. If the boss height is, say, 10 mm, then one should expect that as the knee comes into full extension, then the patella would move laterally by 10 mm since it moves off the boss and onto the anterior cortex of the femur. This would be seen clinically as a J-sign. In this case it would seem to be preferable to perform a deepening trochleoplasty before the MPFL reconstruction, since it would then be easier to define the femoral tunnel position relative to the MPFL origin. Of note is Thanaut and Erasmus’ work [22] describing “favourable anisometry” which emphasised that the primary purpose of the MPFL reconstruction is to stop excessive lateral displacement of the patella and allow the quadriceps muscle to act over the anterior part of the distal femur. The MPFL guides the patella into the trochlea in the first 20° of knee flexion. This can work well if the trochlea is not so dysplastic that there is lateral hypoplasia in its distal portion [3].

The strengths of this study are that the analysis of the groove on lateral radiograph can be performed in a standard clinic. However, it does not give an accurate guide to the tunnel position down to the level of millimetres, nor is it desirable to define a precise tunnel position radiographically pre-operatively as the final position is decided intra-operatively. However the surgeon can use this analysis to decide whether an isolated MPFL reconstruction is a sensible option by noting the severity of any TD and consider how the graft length may change during knee flexion based on the shape of the trochlea. It also shows that the at-risk patient group with patellofemoral instability are different anatomically from the normal and that laboratory studies that use normal cadaveric knees [21, 26] cannot be relied on when treating a patient in a clinic.

This study did not consider the patellar insertion of the MPFL. The effect of patellar height on the MPFL length has been analysed in a computer model [23]. This found that the tension in the MPFL did not change significantly when the Insall-Salvati ratio was between 0.74 and 1.5. It was suggested if the ratio was > 1.5 then the femoral tunnel should be placed more proximally. Others would argue for distalization of the tibial tubercle [24].

For the MPFL to have equal tension throughout flexion within the groove, the length should not change. In normal knees the MPFL does not behave isometrically. The change in length, as measured from Schöttle’s point, was greater for patellofemoral instability patients explaining the finding that an isolated MPFL reconstruction in the presence of severe trochlear dysplasia is contraindicated.

## Conclusion

The centre of the circle formed from the trochlea on lateral plain radiograph was different between control patients and those with patellofemoral instability and did not correlate with the origin of the MPFL as defined by Schöttle’s point for either cohort. The MPFL in the normal knee does not have equal tension throughout flexion but the length change was greater for patellofemoral instability patients and was greatest for those with a boss height ≥ 5 mm. Biomechanical data on the MPFL related to the femoral tunnel position from normal cadavers does not correspond to the patient population at risk of an MPFL reconstruction.
